# Calcium Requirements and Metabolism in Chinese-American Boys and Girls

**DOI:** 10.1002/jbmr.76

**Published:** 2010-03-04

**Authors:** Lu Wu, Berdine R Martin, Michelle M Braun, Meryl E Wastney, George P McCabe, Linda D McCabe, Linda A DiMeglio, Munro Peacock, Connie M Weaver

**Affiliations:** Department of Foods and Nutrition, Department of Statistics, Purdue UniversityWest Lafayette, IN, USA and Indiana University School of MedicineIndianapolis, IN, USA

**Keywords:** asian americans, calcium requirements, calcium kinetics, adolescents

## Abstract

Calcium requirements of North American adolescents were set at 1300 mg/day based on data from white girls. Calcium requirements for Asian-American adolescents have not been studied. Using metabolic balance protocols and a range in calcium intakes, skeletal calcium retention was determined in Chinese-American adolescents. A sample of 29 adolescents, 15 boys aged 12 to 15 years and 14 girls aged 11 to 15 years, was studied twice on paired calcium intakes ranging between 629 to 1835 mg/day using a randomized-order crossover design. Calcium absorption and bone turnover rates using double-stable calcium isotope kinetic analysis on two calcium intakes per subject were measured and compared in boys and girls. Girls and boys had low habitual mean calcium intakes of 648 and 666 mg/day, respectively, and low mean serum 25-hydroxyvitamin D concentrations of 19.1 and 22.2 ng/mL, respectively. True fractional calcium absorption varied inversely with calcium load. Boys had significantly higher bone turnover rate than girls at the same calcium intake. Calcium retention increased with calcium intake; calcium intakes to achieve maximal calcium retention were 1100 mg/day in boys and 970 mg/day in girls. Recommendations for calcium requirements should be lowered for Chinese-American adolescents. © 2010 American Society for Bone and Mineral Research.

## Introduction

The adolescent period is crucial for optimizing future bone health because bone accumulates rapidly during these years and accounts for up to half of adult peak bone mass.(1–3) Calcium intake is critical for adequate bone mineralization. Variations in dietary calcium explained 12.3% of the variation in calcium retention in black and white adolescent girls(4) and 21.7% of the variation in adolescent white boys.(5) The calcium requirement for North American adolescents was based on the minimal calcium intake required to achieve maximal calcium retention, assuming that this level of calcium consumption would optimize development of peak bone mass and reduce the risk of osteoporosis later in life.(6) The adequate intake of calcium for white adolescents aged 9 through 18 years was set as 1300 mg/day based on data in white girls.(7) Calcium requirements set for adolescents in Asian countries are less than the requirement for American adolescents, that is, 1200 mg/day in China, 650 mg/day in Malaysia, and 700 mg/day in Indonesia.(8) However, these calcium requirements were not based on evidence in Asian adolescents. Rather, it was assumed that calcium requirements would be lower for Asian than for white adolescents because of reports that Asians had higher calcium absorption efficiency and higher calcium retention than whites.(9–11) However, those studies did not control dietary intake, and they evaluated calcium absorption at only one calcium intake level. Racial comparisons of calcium absorption between Asian and white American adolescents have not been performed previously in the same laboratory using the same protocol.

Asians currently comprise almost 5% of the American population and are projected to comprise about 8% by 2050.(12) The aim of this study was to measure calcium balance and metabolism as a function of calcium intake in Chinese-American adolescents to provide a basis for establishing calcium requirements for Chinese-American adolescents. Secondary aims were to investigate factors other than calcium intake that affected skeletal calcium retention and to compare calcium kinetics in boys and girls.

## Subjects and Methods

### Subject recruitment

Adolescent boys and girls of Chinese ancestry living in the United States were recruited from Illinois, Indiana, and Michigan. Families responding to mailed flyers were sent a questionnaire to ascertain age in years, race, health, age of onset of menarche for girls, and calcium supplement use of interested volunteers. Boys and girls were eligible if they were between 11 to 15 years of age. Only adolescents who were 100% Asian, as reported by their biological parents based on the heritage of the parents and grandparents, were eligible. Eligibility requirements also included absence of any health problems related to calcium or bone and no current use of calcium supplements. The Institutional Review Boards of Purdue University and Indiana University School of Medicine at Indianapolis approved the study protocol, and informed consent and assent were obtained from the parents and adolescents, respectively.

### Study design

This study used a randomized order, crossover design in which each subject was randomly assigned to one of four paired high-low calcium intake groups representing intake ranging from 600 to 1600 mg/day. The boys and girls were housed in a campus residence hall converted into a metabolic unit for two 3-week balances in the summer separated by a 1-week washout when they returned to their homes. During the balance, participants were scheduled for a variety of educational and recreational activities coordinated as a summer-camp environment. The first 7 days of each balance served as equilibration to the basal diet, and the last 14 days served as the study. All meals, snacks, and beverages were provided, and all urine and feces were collected during the study. Serum samples were collected after an overnight fast at the beginning and end of the first balance and the end of the second balance for measurements of biochemistries. The measures at the end of each balance were used in regression models.

### Bone mineral density and body composition

During the week of equilibration, bone mineral density (BMD) and bone mineral content (BMC) of the femoral neck, total hip, lumbar spine (L_2_ to L_4_), and total body and total-body lean mass, fat mass, and percent body fat were measured using dual-energy X-ray absorptiometry (DXA) (Lunar Corp, Madison, WI, USA). Height and weight were measured by a wall-mounted stadiometer and a calibrated electronic scale, respectively, in light clothing without shoes. Tanner stage maturation(15) was evaluated by a single pediatric endocrinologist (LAD).

### Calcium kinetics

For calcium kinetic studies, on the eighth day of each balance period, a subset of subjects (8 boys and 7 girls) was given a capsule containing 36 mg of ^44^Ca. The capsule was administered orally with breakfast, which had one-third of the daily calcium intake (200 to 666 mg), and a serum sample was collected 60 minutes later. Subjects then were infused with 7.5 mg of ^43^Ca intravenously over 4 minutes, and serum samples were collected at 15 and 30 minutes; 1, 2, 3, 4, 7, and 9 hours; and 1, 2, 3, 5, 7, 9, 11, and 13 days. The orally administered ^44^Ca was used to measure calcium absorption from food, and the intravenous ^43^Ca was used to measure calcium cleared from blood. Serum, urine, and fecal samples were stored for later analysis.

For true fractional ^44^Ca absorption (TFCA) in all subjects not participating in kinetic studies, a single oral isotope of 10 mg ^44^Ca was given as part of a test meal containing one-third of the assigned daily calcium intake. TFCA was calculated by an equation developed for use with a single blood draw 3 hours after oral isotope dose in adolescents.(16)

### Diet

Habitual diet intakes were estimated from 6 day food records collected at home using the Nutrient Data System (Version 5.0-3.5, University of Minnesota, USA). During the balance, a 4-day-cycle menu was used with subjects consuming only the foods and beverages provided. The 4-day-cycle menu was individualized to meet energy needs with cookies that had the same macronutrient proportions as the basic diet. All food and beverages were prepared with deionized water and weighed to the nearest 0.1 g. Beverage glasses were rinsed with deionized water, and the rinse also was consumed. The basal diet provided 500 mg of calcium and approximately 125 IU of vitamin D per day. The varied calcium intake was achieved through daily consumption of 3 cups of orange juice as unfortified and fortified with calcium citrate malate juice to provide the level of assigned calcium, as described previously.(4,7) Subjects drank deionized water *ad libitum*. Duplicate composites of all food and beverages consumed daily were prepared at the same time as the meals, freeze dried, ashed at 600°C, and analyzed for calcium and other minerals in duplicate using inductively coupled plasma spectrophotometry (ICP, Optical Emission Spectrometer, Optima 4300DV; Perkin-Elmer, Shelton, CT, USA).

### Fecal and urine analyses

Urine was collected in acid-washed containers and pooled as 24-hour collections of each day. Total 24-hour urine volume was measured, and the urine sample was acidified with concentrated hydrochloric acid (1% by volume) and frozen at −10°C. Acidified duplicate urine samples were thawed and diluted with 3% HNO_3_ for measurement of calcium content. Fecal samples for the 24 hours were collected in previously weighed containers, weighed, and then immediately frozen. Fecal samples were diluted with concentrated hydrochloric acid and deionized water for homogenization by a stomacher (Tekmar, Cincinnati, OH, USA). Aliquots were sampled in triplicate, dried at 48°C, ashed at 600°C, and diluted with 3% HNO_3_ for measurement of calcium. Calcium content of feces and urine was measured by ICP.

### Biochemistry

Serum collected at the beginning of the balance was analyzed for insulin-like growth factor 1 (IGF-1), insulin-like growth factor binding protein (IGFBP_3_), testosterone, estrone (E1), estradiol (E2), and sex hormone–binding globulin (SHBG) by ELISA (Diagnostic Systems Laboratories, Inc., Webster, TX, USA). Serum collected at the beginning and end of the balance period was measured for parathyroid hormone 1-84 (PTH 1-84) by a two-site immunoassay [coefficient of variation (CV) = 7.1%] (Nichols Institute Diagnostics, San Juan, Capistrano, CA, USA), osteocalcin (OC) by immunoradiometric assay (CV = 6.7%), and bone-specific alkaline phosphatase (BAP) (CV = 4.1%) (Quidel Corp., San Diego, CA, USA), collagen type I cross-linked N-telopeptide (NTX) by ELISA (Ostex International, Seattle, WA, USA), 25-hydroxyvitamin D [25(OH)D], and 1,25-dihydroxyvitamin D [1,25(OH)_2_D] by protein-binding assays (CV = 8.1% and 9.1%, respectively) (DiaSorin, Inc., Stillwater, MN, USA). Baseline serum calcium was part of the screening chemistry profile.

### Collection compliance

Creatinine (Cr) was used to determine urine collection compliance based on constant daily urinary creatinine excretion. It also was used for adjustment to 24-hour periods. Urinary Cr was measured on a Cobas-Mira Plus (Roche Diagnostic Systems, Branchburg, NJ, USA). Corrected daily urinary calcium was calculated as


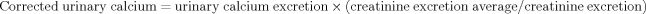
(1)

Polyethylene glycol (PEG, E3350 Dow Chemical Co, Midland, MI, USA), a nonabsorbable fecal marker excreted in the liquid phase of feces, was used to assess fecal collection compliance. Each subject ingested 3 g of PEG every day in six gelatin capsules (two capsules with each meal). Each capsule contained 500 ± 5 mg of PEG. A turbidimetric assay described previously(17) was used to analyze PEG. PEG recovery was used to decide whether data should be included rather than used for fecal Ca adjustment.

### Calcium balance calculation

Fecal samples from the last 2 weeks of each balance were used. A 1-day lag was used when calculating intake minus fecal excretion to accommodate the 19-hour transit time in the gut. Net calcium absorption was calculated as intake minus fecal excretion divided by intake. Calcium retention was calculated as


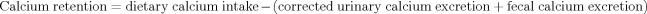
(1)

### Kinetic analysis

Calcium isotope ratios (^44^Ca:^42^Ca and ^43^Ca:^42^Ca) from serum, urine, and feces were determined by inductively coupled plasma spectrophotometry–mass spectrophotometry (ICP-MS).(18) Data from each subject from serum, urine, and feces were fitted by the three-compartment model using the WINSAAM program, as described previously.(19) Subjects were assumed to be in steady state during the last 14 days of each period. *L*_*i*_,_*j*_ is the fraction of compartment *j* moving into compartment *i* per unit time, and *R*_*i*_,_*j*_, transport rate, was calculated as *R*_*i*_,_*j*_ = *L*_*i*_,_*j*_ × *M*_*j*_, where *M*_*j*_ is the day mass of compartment *j*. Bone turnover rate comprises bone formation *V*_0+_ and bone resorption *V*_0_–. Calcium absorption α, urinary calcium excretion, fecal calcium excretion, and endogenous fecal calcium excretion were determined. Bone deposition is determined from the final slope of the exponential curve so that fractional loss is multiplied by the mass of the lowest exchangeable pool. Bone resorption is the rate of calcium release from bone and is determined as the calcium required to enter blood from bone to maintain a constant pool size.


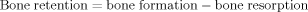
(3)

Calcium absorption was calculated as the fraction of calcium entering the serum from the intestine.(19)

### Statistical analysis

Two-sample *t* tests were used to compare the means of boys and girls on various measures. The relationships between fecal calcium excretion and calcium intake and between urine calcium excretion and calcium intake were approximated by linear functions in models that included gender and the interaction between gender and intake. A data-smoothing procedure was used to describe the relationship between calcium intake and calcium retention for girls and boys separately. This led to identification of a parametric model that provided a similar fit to the intake and retention data. This function then was estimated using nonlinear regression. A bootstrap method that takes into account the fact that subjects can provide data at different intakes was used for statistical inference. Residuals from the nonlinear models were used to examine the possible contribution of additional variables. A parametric model that combined data from both genders was used to estimate the effect of gender on the relationship between calcium intake and calcium retention. The relationship between fecal calcium excretion and calcium intake was described by a smooth function. Statistical inference was performed using a linear approximation to the relationship. The same approach was used for urinary calcium excretion. All statistical analyses were performed using SAS software (Version 9.2, SAS Institute, Cary, NC, USA).

## Results

### Subjects

Subjects were 15 healthy boys and 14 healthy girls, 28 Chinese and one Chinese-Vietnamese, who had been in the United States at least 8 months (Table 1). All but one boy completed the first balance, and all completed the second. A subset of 8 boys and 7 girls completed the kinetic studies during both balances.

**Table 1:** Baseline Characteristics of Chinese-American Subjects

	Boys (*n* = 15), Mean ± SD		Girls (*n* = 14), Mean ± SD	
		
	Min	Max	Min	Max
Age (years)	14.0 ± 1.0	12.6–15.5	13.3 ± 1.3	11.2–15.7
Height (cm)	162.2 ± 10.2	145.7–177.1	156.5 ± 7.1	138.9–165.1
Weight (kg)	55.4 ± 14.6	31.2–82.7	47.1 ± 8.6	36.2–62.8
BMI (kg/m^2^)	20.8 ± 4.1	14.7–29.4	19.2 ± 2.5	15.9–24.7
Tanner score	3.5 ± 1.6	1.0–5.0	3.2 ± 1.4	1.0–5.0
PMA (months)			13.9 ± 21.3	0.0–71.0
Total-body BMC (g)	2100 ± 534	1150–2902	1877 ± 337	1347–2546
Total-body BMD (g/cm^2^)	1.04 ± 0.10	0.83–1.18	1.02 ± 0.05	0.95–1.11
Habitual calcium intake (mg/day)	666 ± 349	78–1305	648 ± 321	160–1155
SHBG (nmol/L)	39.8 ± 27.7	10.9–119.4	52.6 ± 30.0	8.0–108.1
Testosterone (ng/mL)	3.7 ± 1.9	1.1–6.5		
E1 (estrone) (pg/mL)			50.4 ± 26.1	12.1–114.3
E2 (estradiol) (pg/mL)			35.1 ± 16.2	3.2–62.7
1,25-Dihydroxyvitamin D (pg/mL)	74.4 ± 30.8	39.4–156.1	59.9 ± 10.5	41.2–72.9
25-Hydroxyvitamin D (ng/mL)	22.2 ± 5.7	13.8–36.2	19.1 ± 5.9	13.2–36.2
IGF-1 (ng/mL)	478.7 ± 117.1	320.1–703.8	476.6 ± 110.2	119.9–611.4
IGFBP_3_ (ng/mL)	5743 ± 730	4395–6665	6134 ± 1165	3841–8058
Serum PTH (pg/mL)*	27.6 ± 8.9	12.5–42.4	41.2 ± 18.7	14.6–81.8
Serum osteocalcin (ng/mL)	27.7 ± 5.9	18.3–38.1	22.8 ± 10.4	4.6–41.9
Serum BAP (ng/mL)*	80.3 ± 22.1	34.6–112.2	56.6 ± 28.9	16.8–110.7
Total alkaline phosphatase (U/L)*	254.9 ± 78.2	102.0–373.0	181.1 ± 94.6	61.0–385.0
Serum NTX (nmol BCE)*	116.9 ± 45.4	35.0–199.4	69.1 ± 52.9	14.7–232.7
Serum calcium (mg/dL)*	9.5 ± 0.2	9.1–9.7	9.6 ± 0.3	9.2–10.2

* Differences *p* < .05,

#### Anthropometrics, habitual calcium intake, and biochemistry

The girls had similar age, height, weight, body mass index (BMI), sexual maturity, and total body bone mineral content (BMC) as the boys, and the average habitual calcium intake was 666 mg/day for boys and 648 mg/day for girls. Girls had significantly lower fasting serum bone-formation markers, total serum alkaline phosphatase, and bone-specific alkaline phosphatase (BAP) and lower bone resorption marker serum NTX than boys. Girls had significantly higher serum fasting PTH and lower serum calcium levels than boys. There was no difference in SHBG, 1,25-dihydroxyvitamin D, 25-hydroxyvitamin D, IGF-1, IGFBP3, and osteocalcin.

### Calcium balance

Calcium intakes ranged from 629 to1220 mg/day for the lower calcium balance and from 1274 to 1835 mg/day for the higher calcium balance. There was no significant difference between the uncorrected urinary calcium and corrected urinary calcium (*p* > .05). Fecal collection compliance measured by PEG recovery averaged 83.4%.

**Fig. 1:**
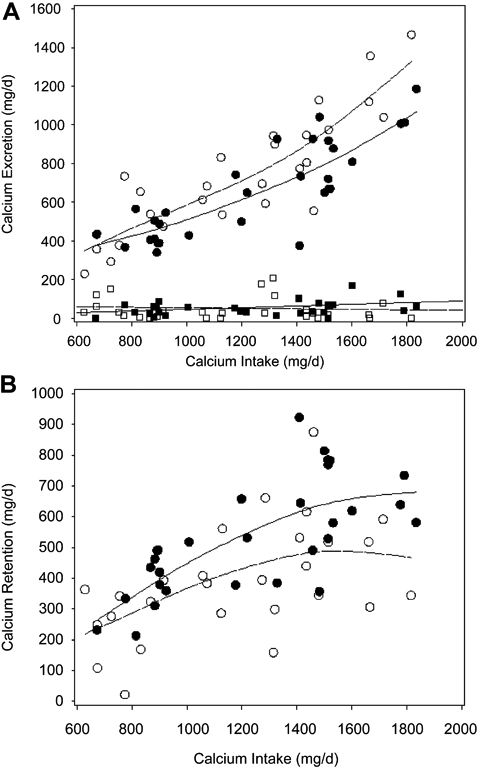
Calcium intake and (*A*) calcium excretion or (*B*) retention in Chinese-American adolescents (girls: *open symbols* and *dashed lines*; boys: *closed symbols* and *solid lines*). In panel *A*, fecal is circles and urine is squares.

Fecal calcium increased with calcium intake for both boys (*p* < .001) and girls (*p* < .001), and the increase was more rapid for girls than for boys (one-sided *p* = .043; Fig. 1*A*). Urinary calcium increased for both boys (*p* <. 01) and girls (*p* < .02), and there was no difference in the slopes (*p* = .81; Fig. 1*A*). Calcium retention increased with calcium intake (*p* < .002), with boys retaining more calcium than girls (*p* = .02; Fig. 1*B*).

### Statistical modeling (Fig. 2)

To establish mean maximal retentions, the relationship between calcium intake and calcium retention was described by a nonlinear regression model *Y* = β_0_*e*^*L*^/(1 + *e*^*L*^), where *Y* is the mean calcium retention for a given intake, *L* equals β_1_ + β_2_*X*, and *X* is calcium intake (mg/day). In this model, β_0_ is the retention for an arbitrarily large intake. The fitted models for boys and girls were


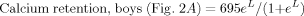


where *L* = −3.11 + 0.00371(intake), and


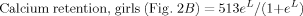


where *L* = −2.54 + 0.00346(intake).

**Fig. 2:**
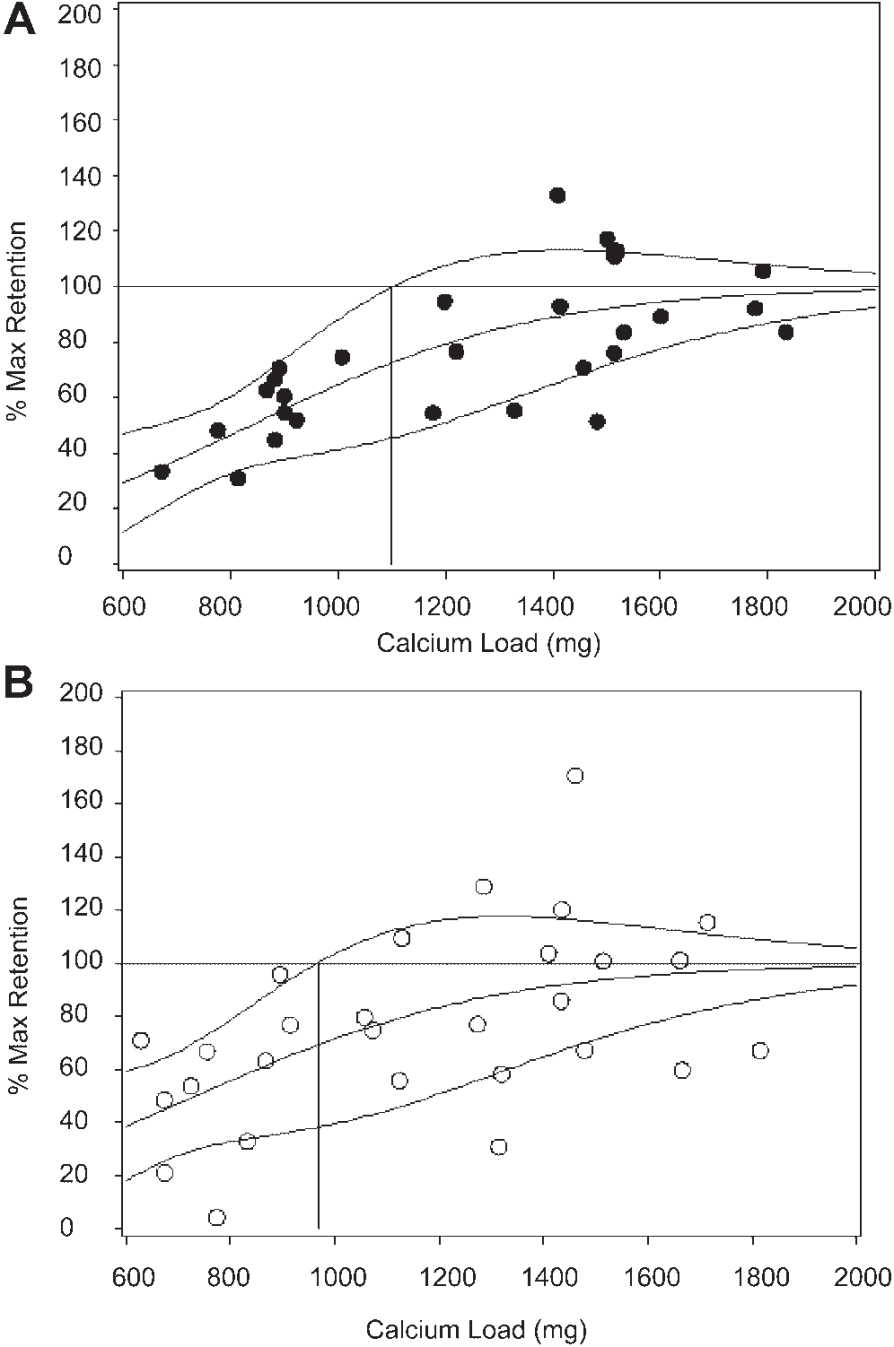
Maximal calcium retention as a function of calcium intake (the three lines represent means and 95% confidence intervals resulting from the nonlinear model) in Chinese-American adolescent boys (*A*) and girls (*B*).

The coefficient for calcium intake was statistically significant for both boys (*p* < .05) and girls (*p* < .05). The mean maximal calcium retention (the estimate of β_0_ in these models) for boys was 695 ± 83 mg/day, and the mean maximal calcium retention for girls was 513 ± 82 mg/day (one-sided *p* < .06). The calcium intake for maximal retention defined as the lowest intake for which the confidence interval for retention includes the mean maximal calcium retention was 1110 mg/day for boys and 970 mg/day for girls. Calcium intake explained 29% of the variation in calcium retention in girls and 53% in boys.

Residuals from these nonlinear models were used to examine the ability of measures other than calcium intake to explain additional variability in calcium retention. These included age, height, weight, Tanner score, total-body BMD, total-body BMC, postmenarcheal age (PMA), IGF-1, IGFBP3, estrone, E2, SHBG, testosterone, PTH, 25(OH)D, 1,25(OH)D_3_, total alkaline phosphatase, BAP, and OC. (Measures by individual are in the online appendix). In girls, E2 explained an additional 15% (negative relationship) of the variation in calcium retention. In boys, additional variation (positive relationships) in calcium retention was explained by testosterone (13%), Tanner stage (8%), and height (8%), but the effects were not additive because these explanatory variables were correlated.

The model for boys and girls combined was


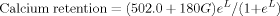


where *L* = −2.91 – 0.360*G* + 0.00398(intake), and *G* = 1 for males and 0 for females.

### Kinetic studies

**Fig. 3:**
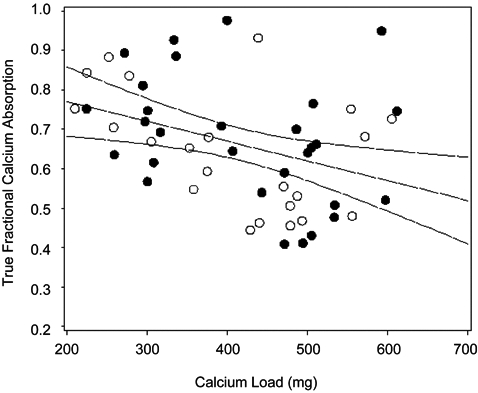
Relationship of calcium load and fractional calcium absorption in adolescent Chinese-American boys and girls (*r* = −0.37, *p* = .002).

TFCA was inversely related to calcium load (*r* = −0.37, *p* = .0002) and similar between boys and girls, so they were combined (Fig. 3). In statistical models that accounted for session, sex, and multiple observations in the same individuals, there were no significant (*p* < .05) relationships between TFCA and fasting PTH, or 25(OH)D, or 1,25(OH)_2_D_3_ levels. In the subset of adolescents who underwent kinetic studies, daily calcium intakes ranged from 629 to 1008 mg/day for the lower calcium balance and from 1274 to 1602 mg/day for the higher calcium balance. When adjusted for calcium intake (629 to 1515 mg/day), boys had significantly higher (*p* < .01) bone-formation rates and significantly higher (*p* < .01) bone-resorption rates than girls (Table 2).

**Table 2:** The Kinetics of Chinese-American Adolescent Girls and Boys

	Boys**	Girls**
	
	Mean ± SE*	Mean ± SE*
Fractional absorption from breakfast	0.578 ± 0.048	0.599 ± 0.044
Fractional absorption from daily diet	0.554 ± 0.020	0.542 ± 0.019
Absorbed calcium (mg/day)	662.4 ± 29.9	567.1 ± 27.3
Bone-formation rate (mg/day)^a^	2416.3 ± 94.6	1368.5 ± 86.4
Bone-resorption rate (mg/day)^a^	1986.3 ± 97.3	992.0 ± 88.8
Urinary calcium excretion (mg/day)	78.4. ± 6.0	87.0 ± 5.5
Fecal calcium excretion (mg/day)	702.2 ± 20.4	604.3 ± 18.6
Endogenous excretion (mg/day)	154.0 ± 18.9	103.7 ± 17.2
Bone balance (mg/day)	430 ± 24	376 ± 22
Calcium intake (mg/day)	1211 ± 130	1068 ± 118

* Adjusted for order, session, and calcium intake (high/low).

** Eight boys contributed 15 observations and 7 girls contributed 14 observations.

a *p* < .05 boys > girls.

**Fig. 4:**
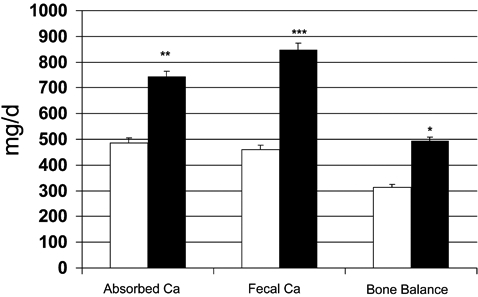
Significant differences in calcium kinetic parameters owing to calcium intake in a subset of 8 boys and 7 girls. Open bars are lower intakes (767 ± 40 mg/day for girls or 907 ± 77 mg/day for boys), and solid bars are on higher calcium intakes (1374 ± 30 mg/day for girls and 1500 ± 55 mg/day for boys). *p < .05; **p < .01; ***p < .001.

There were no differences in rates of urinary excretion, bone resorption, bone formation, and endogenous calcium excretion at lower and higher calcium intakes. However, there were significant positive calcium intake effects on total absorbed calcium (*p* < .01), fecal calcium excretion (*p* < .001), calcium retained in bone (*p* < .05), and the ratio of bone formation to bone resorption (*p* < .05; Fig. 4). Biochemical markers of bone turnover did not predict bone turnover rates determined by calcium kinetics.

## Discussion

We investigated calcium requirements of Chinese-American boys and girls. Chinese-American adolescent girls had similar anthropometric characteristics as boys in our sample, including age, height, weight, Tanner score, and total-body BMD and BMC. At low calcium intakes, the retentions for boys and girls were similar, but at high calcium intakes, boys had 180 ± 66 mg/day higher retention than girls. The difference between Chinese-American boys and girls at higher calcium intakes is similar to the sex difference of 171 ± 38 mg/day calcium retention of white adolescents reported previously,(20) but in the white adolescents, the difference was constant over the full range of calcium intakes studied, that is, 700 to 2200 mg/day. Boys retained significantly more calcium at higher calcium intakes than girls through higher net calcium absorption.

Although it has been assumed that calcium requirements would be lower in Asians than in whites owing to greater calcium absorption efficiency,(9–11) this had not been directly tested. Assessing calcium intake for maximal calcium retention was a main approach for determining calcium requirements by the Institute of Medicine (IOM).(6) We used the same nonlinear model to determine maximal calcium retention as developed for white adolescents.(7) The mean maximal calcium retention for Chinese-American boys was 695 mg/day, and the mean maximal calcium retention for girls was 513 mg/day, both of which were higher than the reported values in white adolescent boys and girls (629 and 473 mg/day, respectively.(7,20) Although the white boys and girls were studied in different years than the Chinese Americans, the protocols were the same. The minimal calcium intake leading to the maximal calcium retention was 1110 mg/day for Chinese-American boys and 970 mg/day for Chinese-American girls. The value for Chinese-American boys was somewhat lower thano that for white boys (1140 mg/day),(20) but the value for Chinese-American girls was considerably lower than that for white girls (1300 mg/day).(7) Thus our data suggest that the calcium requirement for Chinese-American girls is lower than the recommendation for white adolescent girls.(6) This increased efficiency was related to increased calcium absorption efficiency and renal calcium conservation. Consistent with other studies,(10,21) our Chinese-American adolescents had low urinary calcium excretion (mean 53.8 mg/day) compared with white girls (mean 93.9 mg/day).(22)

Chinese-American boys and girls had a significant inverse relationship between TFCA and calcium load under a wide range of calcium loads, as has been reported previously in white adults(23) and Chinese children,(9) but not white girls.(19) As calcium load increased, total calcium absorbed increased, that is, 160 mg at a load of 200 mg, 280 mg at a load of 400 mg, and 360 mg at a load of 600 mg. TFCA was not influenced by fasting PTH or 1,25(OH)_2_D_3_. Compared with white girls,(19) Chinese-American girls had higher calcium absorption efficiency on comparable calcium loads, especially at lower calcium loads. The higher calcium utilization efficiency and lower calcium requirements we observed in Chinese-American adolescents may relate to their lower habitual calcium intakes, lower vitamin D status, smaller body size, higher absorption efficiency, and different lifestyles, including physical activity. Compared with white adolescents [serum 25(OH)D 29 ng/mL for white boys and 34 ng/mL for white girls] examined in our previous metabolic studies,(4,20) this sample of Chinese-American adolescents had significantly lower vitamin D status. These data agree with serum 25(OH)D levels in Asians reported in other studies.(9,21) This sample of Chinese-American adolescents also had significantly lower habitual calcium intake than white adolescents, as has been reported by others.(9–11,24,25) Our subjects ate a mixture of traditional Chinese foods and Western foods that included higher dairy product consumption than adolescents living in China.

Calcium intake was the largest predictor of calcium retention that we evaluated. Sex steroid hormones explained an additional almost 15% of the variability in calcium retention in girls. The relationship was negative, reflecting epiphaseal closure as estrogen levels become sufficiently high and linear growth ceases.(26) In white boys, serum IGF-1 was a stronger predictor of calcium retention than testosterone, but these measures were highly correlated.(5)

Our calcium kinetic studies showed that Chinese-American boys had significantly higher bone-formation and bone-resorption rates than Chinese American girls across a wide range of calcium intakes. Biochemical markers of bone turnover supported higher bone turnover rates in boys than in girls. Using full kinetic modeling, Wastney and colleagues(19) found that when white girls were on a high-calcium intake, neither fractional calcium absorption nor bone-formation rate changed, but total absorbed calcium and urinary calcium excretion increased, whereas bone-resorption rate decreased. Chinese-American girls had greater fractional absorption efficiency that was more responsive to calcium intake compared with white girls on similar calcium loads (Chinese intake: 767 to 1374 mg/day, α = 0.60 to 0.58; white intake: 860 to 1900 mg/day, α = 0.48 to 0.48). When calcium intakes increased from a low to an adequate level, bone-resorption rate decreased in white girls (intake: 860 to 1900 mg/day; *V*_0_– = 1400 to 960 mg/day), whereas no significant changes were observed in Chinese-American girls (intake: 767 to 1374 mg/day; *V*_0_– = 930 to 996 mg/day). The bone-resorption rate in Chinese girls was already as low on the low calcium intakes as for the white girls on high calcium intakes. Since bone-formation rates were higher in white than in Chinese-American girls and did not change with calcium intake in either group, bone balance was higher in Chinese-American girls than in white girls on low calcium intake but not on higher calcium intakes (Chinese-American girls' intake: 767 to 1374 mg/day, *V*_0+_ = 1208 to 1454 mg/day, bone balance: 278 to 458 mg/day; white girls intake: 860 to 1900 mg/day, *V*_0+_ = 1528 to 1536 mg/day, bone balance: 130 to 580 mg/day). Thus skeletal calcium accretion increases with calcium intake but peaks at a lower level in Chinese-American than in white girls. Calcium intake effects on calcium kinetic parameters were not statistically different for boys and girls, but there were sex differences in bone turnover rates in contrast to a study in prepubertal children.(27)

This study had many strengths. It was a controlled-feeding crossover study. We studied calcium metabolism across a wide range of calcium intake levels. Boys and girls were studied together so that we could directly compare sex differences. The weakness is the relatively small sample of Chinese-Americans, limiting the generalizability to other Asian populations.

Calcium retention increased with calcium intake to a maximal calcium retention of 695 and 513 mg/day at intakes of 1110 and 970 mg/day for Chinese-American adolescent boys and girls, respectively. Calcium intake explained much of the variability in calcium retention (53% in boys and 29% in girls). Sex steroid hormones explained an additional 15%. Boys retained significantly more calcium than girls as calcium intake increased through lower fecal excretion and higher net absorption. Racial differences in calcium metabolism and retention explain much of racial differences in bone mass.
